# Protein tyrosine phosphatase receptor type D gene promotes radiosensitivity via STAT3 dephosphorylation in nasopharyngeal carcinoma

**DOI:** 10.1038/s41388-021-01768-8

**Published:** 2021-04-06

**Authors:** Yanling Lin, Xiaohan Zhou, Kaifan Yang, Yuting Chen, Lingzhi Wang, Wenxiao Luo, Yujiang Li, Jinrong Liao, Yingtong Zhou, Yiming Lei, Yanting Zhang, Dehua Wu, Longmei Cai

**Affiliations:** 1 Department of Radiation Oncology, Nanfang Hospital, Southern Medical University, Guangzhou, China; 2 Division of Spine Surgery, Department of Orthopaedics, Nanfang Hospital, Southern Medical University, Guangzhou, China; 3 First Clinical Medical College, Nanfang Hospital, Southern Medical University, Guangzhou, China; 4 Department of Thoracic and Cardiovascular Surgery/Huiqiao Medical Center, Southern Medical University, Guangzhou, China; 5 Department of Thoracic and Cardiovascular Surgery, Affiliated Dongguan People’s Hospital, Southern Medical University (Dongguan People’s Hospital), Dongguan, China; 6 Second Clinical Medical College, Zhujiang Hospital, Southern Medical University, Guangzhou, China

**Keywords:** Radiotherapy, Prognostic markers

## Abstract

Radiotherapy is essential to the treatment of nasopharyngeal carcinoma (NPC) and acquired or innate resistance to this therapeutic modality is a major clinical problem. However, the underlying molecular mechanisms in the radiation resistance in NPC are not fully understood. Here, we reanalyzed the microarray data from public databases and identified the protein tyrosine phosphatase receptor type D (*PTPRD*) as a candidate gene. We found that *PTPRD* was downregulated in clinical NPC tissues and NPC cell lines with its promoter hypermethylated. Functional assays revealed that *PTPRD* overexpression sensitized NPC to radiation in vitro and in vivo. Importantly, miR-454-3p directly targets *PTPRD* to inhibit its expression and biological effect. Interestingly, mechanistic analyses indicate that *PTPRD* directly dephosphorylates STAT3 to enhance Autophagy-Related 5 *(ATG5)* transcription, resulting in triggering radiation-induced autophagy. The immunohistochemical staining of 107 NPC revealed that low *PTPRD* and high p-STAT3 levels predicted poor clinical outcome. Overall, we showed that *PTPRD* promotes radiosensitivity by triggering radiation-induced autophagy via the dephosphorylation of STAT3, thus providing a potentially useful predictive biomarker for NPC radiosensitivity and drug target for NPC radiosensitization.

## Introduction

Nasopharyngeal carcinoma (NPC), a malignancy derived from the nasopharyngeal epithelium, has a particularly high prevalence in Southern China and Southeast Asia [1]. The etiologic factors for NPC include Epstein–Barr virus (EBV) infection, genetic susceptibility, and environmental factors, such as the consumption of food with volatile nitrosamines [2]. According to the NCCN guidelines, early-stage NPC patients should receive radiotherapy (RT) as the standard regimen, whereas those with locally advanced NPC should receive concurrent chemoradiotherapy [3]. Despite the development of high-precision RT techniques, a significant percentage of patients develop resistance to RT, thus causing treatment failure [4]. Further, the identification and therapy of radioresistant NPC remain a clinical problem. Therefore, revealing the mechanisms underlying radioresistance is important to develop novel strategies for enhancing the efficacy of RT in NPC patients.

Protein tyrosine phosphatases play a vital role in regulating cancer cellular functions, such as cell proliferation, adhesion, and apoptosis [5]. Particularly, the PTP receptor-type D (PTPRD) has been reported to be a tumor suppressor. Previous studies have demonstrated that PTPRD is downregulated by genetic (homozygous deletion, loss-of-function mutation, and copy number loss) and epigenetic (miRNA and methylation) modifications in different types of human cancers, including glioblastoma, colon cancer, breast cancer, lung cancer, and head and neck squamous cell carcinoma [6–11]. PTPRD cooperates with CD44 and the β-catenin/TCF signaling to regulate cell migration in colon cancer [7]. Additionally, another study has demonstrated that phosphorylated STAT3 (p-STAT3) is a substrate of PTPRD, and that the downregulation of PTPRD enhances stemness and promotes migration and invasion via the Jak/STAT3 pathway in breast cancer [8]. However, only a few studies have explored the molecular function and detailed mechanisms of PTPRD and its role in NPC.

Autophagy is a highly conserved catabolic process that degrades and recycles damaged proteins and organelles in response to cellular stress, such as starvation and hypoxia [12–14]. Autophagy plays a double-edged sword role in cancer [15]. In NPC, Zhu et al. have showed that the inhibition of autophagy by miR-106A-5p promotes a malignant phenotype [16]. Furthermore, Liu et al. have reported that the TIPE1‐mediated autophagy suppression promotes NPC proliferation via the AMPK/mTOR signaling pathway [17]. ANXA6 has also been shown to contribute to radioresistance in NPC by promoting autophagy via the inhibition of the PI3K/AKT/mTOR pathway [18]. In another study, the mTOR inhibitor enhanced the radiosensitivity of NPC cells by activating autophagy and apoptosis [19]. Overall, the role of autophagy in anticancer therapeutics remains controversial.

In this study, we investigated the mechanism underlying NPC radiosensitivity, identified a novel substrate, and biological function of PTPRD, and revealed a targetable pathway to sensitize NPC to radiation.

## Results

### PTPRD is hypermethylated and downregulated in clinical NPC tissues

**Fig. 1:**
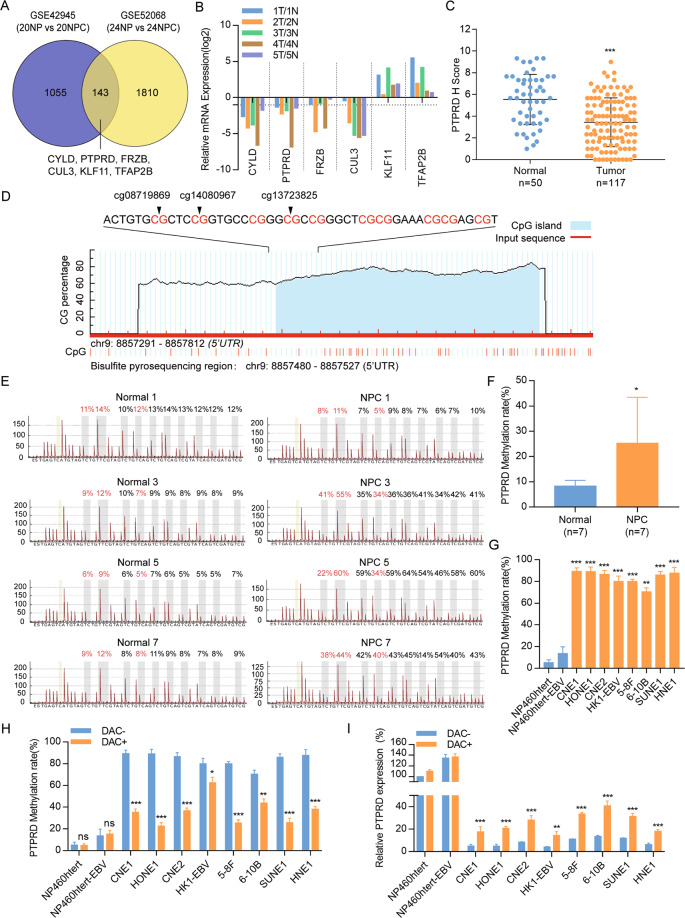
PTPRD is downregulated and hypermethylated in NPC. **A** Venn Diagram: identification of the differentially expressed genes and differentially methylated genes in NPC and noncancerous nasopharyngeal tissues using two microarray data sets. The blue circles represent GSE42945, whereas yellow circles represent GSE52068. **B** Real-time RT-PCR analysis of six candidate genes in the five-paired primary samples (1N, 1T, 2N, 2T, 3N, 3T, 4N, 4T, 5N, and 5T). **C** IHC statistical analysis of *PTPRD* expression in 117 NPC tissues and 50 NP tissues. Mean ± SD; Student’s *t* tests; ***P < 0.001. **D** Schematic illustration of the PTPRD promoter CpG islands and the bisulfite pyrosequencing region. TSS transcription start site, blue region: CpG islands; red region: input sequence; magenta words: CG sites for bisulfite pyrosequencing. **E** Bisulfite pyrosequencing analysis of the PTPRD promoter region in NPC tissues and normal tissues. Magenta words: CG sites of cg08719869, cg14080967, and cg13723825. **F** Average methylation levels in seven pairs of normal and NPC tissues. Mean ± SD (*n* = 7); Student’s *t* tests; *P < 0.05. **G** Bisulfite pyrosequencing analysis of the PTPRD promoter region in two normal nasopharyngeal epithelial cell lines (NP460htert and NP460htert-EBV) and six NPC cell lines (CNE1, HONE1, CNE2, HK1-EBV, 5–8F, 6–10B, SUNE1, and HNE1). These data are representative of three independent experiments. Mean ± SD; Student’s *t* tests; **P < 0.01; ***P < 0.001. **H**, **I** *PTPRD* methylation levels measured via bisulfite pyrosequencing analysis and relative *PTPRD* mRNA levels measured via real-time RT-PCR analysis with (DAC+) or without (DAC−) DAC treatment in NP and NPC cell lines. Mean ± SD; Student’s *t* tests; *P < 0.05; **P < 0.01; ***P < 0.001. These data are representative of three independent experiments.

To identify the potential NPC-specific suppressor regulated by epigenetic aberrations, we reanalyzed the previous miRNA microarray (GSE42945) and the methylation microarray (GSE52068) data. On the one hand, we have previously identified 69 miRNAs that were differentially expressed in NPC (*n* = 20) and noncancerous nasopharyngeal (NP) samples (*n* = 20) [20]. Through TargetScan and miRanda algorithms, 1198 genes were predicted to downregulated in NPC. On the other hand, there were 1953 differentially methylated genes [21]. Thus, 143 common genes were identified to be downregulated by the miRNA and hypermethylated in methylation microarrays (Fig. 1A).

Kyoto Encyclopedia of Genes and Genomes pathway and Gene Ontology (GO) functional analyses were performed to further explore the potential mechanism of these genes (Fig. S1A, B). After hierarchical clustering, top six differentially expressed genes were identified in five NPC, and five noncancerous nasopharyngeal samples and confirmed using RT-qPCR. They included *CYLD, PTPRD, FRZB, CUL3, KLF11*, and *TFAP2B* (Fig. 1B). We focused on *PTPRD* because its protein level was significantly decreased in 117 NPC tissues compared with 50 NP tissues using IHC (Figs. 1C, S1C).

To confirm the hypermethylated status of *PTPRD*, bisulfite pyrosequencing analysis was performed in the other NPC (*n* = 7) and NP specimens (*n* = 7). The selected regions for bisulfite pyrosequencing include three hypermethylated CpG sites (cg08719869, cg14080967, and cg13723825) and are shown in Fig. 1D. The methylation levels of *PTPRD* were significantly upregulated in NPC tissues compared with normal tissues, indicating that *PTPRD* is hypermethylated in NPC (Figs. 1E, F, S2A). Consistently, the genome-wide methylation microarray data downloaded from GEO (GSE62336 and GSE52068) found that the methylation level of *PTPRD* was higher in NPC tissues than NP tissues (Fig. S2B). In addition, the methylation levels of PTPRD in two normal nasopharyngeal epithelial cell lines were significantly lower than eight NPC cells (Figs. 1G, S2C). To investigate whether the hypermethylation of PTPRD caused its downregulation, the demethylation drug DAC was used. Following DAC treatment, PTPRD methylation levels were clearly decreased in the NPC cell lines (Fig. 1H), and PTPRD mRNA levels were substantially increased (Fig. 1I).

### PTPRD overexpression sensitizes NPC cells to radiation

**Fig. 2:**
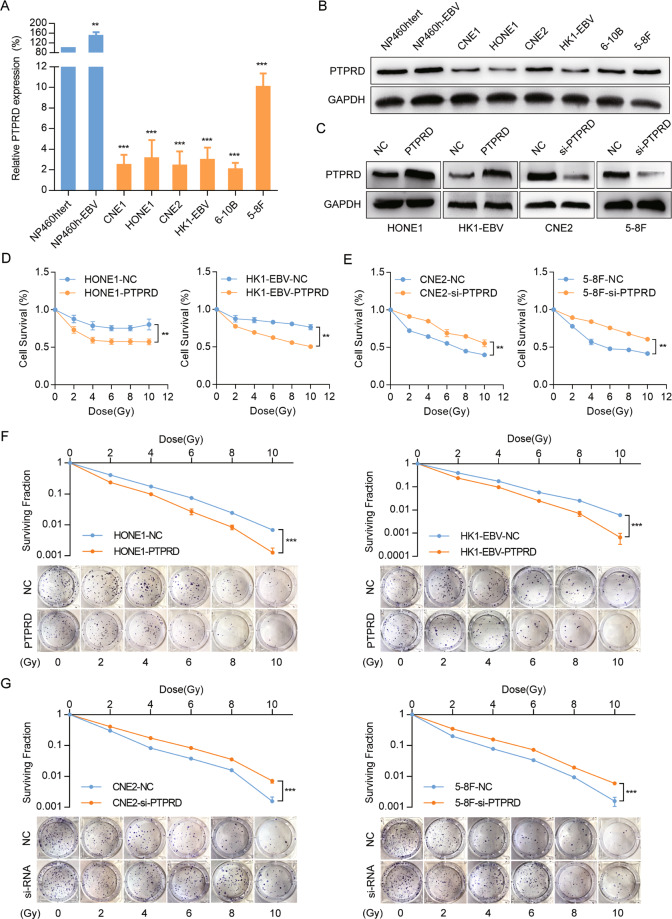
Overexpression of *PTPRD* increases NPC cell sensitivity to radiation. **A**, **B** qRT-PCR analysis of PTPRD mRNA expression and western blotting analysis of PTPRD protein expression in two normal nasopharyngeal epithelial cell lines (NP460hTert-EBV, NP460hTert) and six NPC cell lines (CNE1, CNE2, HONE1, 5–8F, 6–10B, and HK1-EBV). GAPDH was used as an internal control. These data are representative of three independent experiments. Mean ± SD (*n* = 3); Student’s *t* tests; **P < 0.01; ***P < 0.001. **C** Effects of *PTPRD* overexpression and knockdown were confirmed by western blotting. These data are representative of three independent experiments. **D**, **E** CCK8 assay were performed to evaluate cell survival in *PTPRD*-knockdown and -overexpressing cells after exposure to the indicated radiation doses (0, 2, 4, 6, 8, 10 Gy) and culture for 48 h subsequently. These data are representative of three independent experiments. Mean ± SD (*n* = 5); two-way ANOVA and Tukey multiple comparison tests; **P < 0.01; ***P < 0.001. **F**, **G** Colony formation assay were performed to evaluate cell colony forming ability in *PTPRD*-knockdown and -overexpressing cells after exposure to the indicated radiation doses (0, 2, 4, 6, 8, 10 Gy) and culture for 10 days subsequently. These data are representative of three independent experiments. Mean ± SD (*n* = 3); two-way ANOVA and Tukey multiple comparison tests; **P < 0.01; ***P < 0.001.

We performed RT-PCR and western blotting analyses to detect the mRNA and protein levels of *PTPRD* in two normal nasopharyngeal epithelial cell lines and six NPC cell lines. Both the mRNA (Fig. 2A) and protein (Fig. 2B) levels of PTPRD were significantly downregulated in the NPC cell lines. Subsequently, we generated HONE1 and HK1-EBV cells stably expressing *PTPRD* or the control vector, whereas *PTPRD-*knockdown in CNE2 and 5–8F cells with PTPRD-specific siRNA oligos. The infection and transfection efficiency were validated using RT-PCR or western blotting assays (Figs. 2C, S3).

To determine whether *PTPRD* could increase radiation sensitivity in NPC, we evaluated the cell survival in *PTPRD*-knockdown and -overexpressing cell lines via Cell Counting Kit-8 (CCK8) and colony formation assays. *PTPRD*-overexpressing NPC cells exposed to radiation (0–10 Gy) showed a significant reduction in survival compared with the control (Fig. 2D). In contrast, the cells transfected with *PTPRD*-specific siRNA oligos and exposed to radiation demonstrated significantly increased survival compared with the control (Fig. 2E). Consistent with these results, colony formation assay showed that *PTPRD* overexpression resulted in the radiation sensitization of HONE1 and HK1-EBV cell lines. The clonogenic potential of *PTPRD*-overexpressing cells after radiation exposure was less than that of the control NPC cells (Fig. 2F). Reciprocally, we observed increased clonogenic potential in CNE2 and 5–8F cells upon *PTPRD* knockdown and irradiation (Fig. 2G). These results indicate that *PTPRD* overexpression increases radiation sensitivity in NPC cells.

### miR-454-3p directly targets PTPRD and regulates radiation sensitivity in NPC cells

**Fig. 3:**
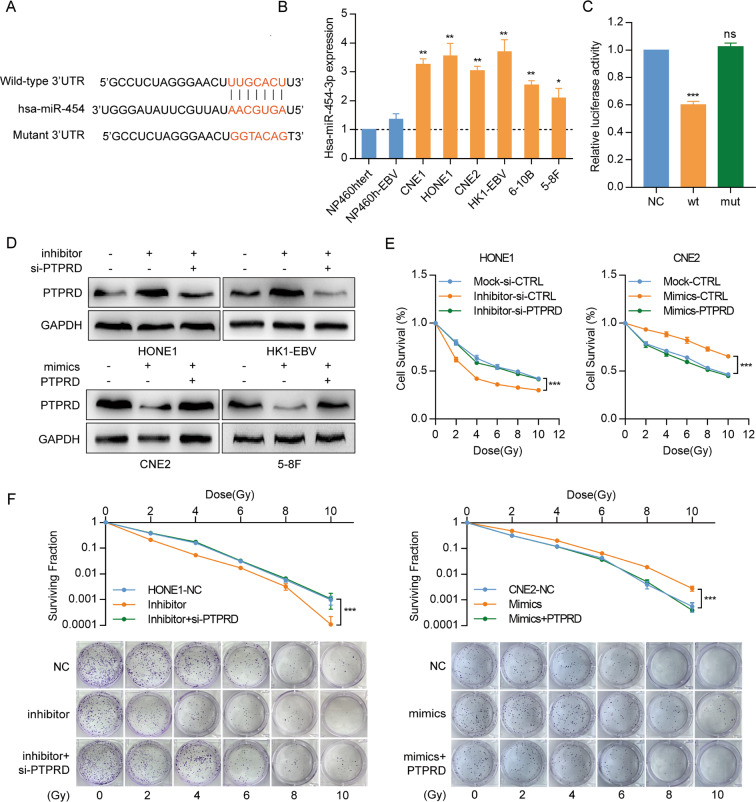
miR-454-3p directly targets *PTPRD* and regulates radiation sensitivity in NPC cells. **A** Bioinformatics predictions of a binding site by miR-454-3p in the *PTPRD* 3′UTR region. Wild-type (wt) and mutant (mut) sequences are indicated. **B** qRT-PCR analysis of miR-454-3p mRNA expression in normal nasopharyngeal epithelial and NPC cell lines. These data are representative of three independent experiments. Mean ± SD (*n* = 3); Student’s *t* tests; *P < 0.05; **P < 0.01. **C** Luciferase reporter assays. 293T cells were co-transfected with the wild-type or mutated *PTPRD* 3′UTR and the miR-454-3p mimics or nonspecific mimic control (NC). Relative repression of firefly luciferase expression was standardized to a transfection control. These data are representative of three independent experiments. Mean ± SD (*n* = 3); Student’s *t* tests; ***P < 0.001; **D** *PTPRD* expression was detected following transfection with miR-454-3p inhibitor or co-transfection with inhibitor and *PTPRD*-specific siRNA oligos in HONE1 and HK1-EBV cells, as well as following transfection with miR-454-3p mimics or co-transfection with mimics and *PTPRD*-specific plasmid in CNE2 and 5–8F cells. GAPDH was used as a loading control. These data are representative of three independent experiments. **E**, **F** CCK8 and colony formation assays were performed to evaluate cell survival after radiation exposure (0, 2, 4, 6, 8, and 10 Gy) and culture cells for 48 h and 10 days respectively. These data are representative of three independent experiments. Mean ± SD (*n* = 5 and *n* = 3 respectively); two-way ANOVA and Tukey multiple comparison tests; ***P < 0.001.

Through TargetScan and RNAhybrid algorithms, *PTPRD* was predicted to be a direct target of miR-454-3p, one of the miRNA previously identified in our microarray study (Fig. 3A). To determine whether miR-454-3p regulates *PTPRD* expression in NPC cells, we first examined its expression in NP and NPC cell lines. miR-454-3p expression was elevated in NPC cells but was weakly expressed in the two normal nasopharyngeal epithelial cell lines (Fig. 3B). Subsequently, miR-454-3p inhibitors were introduced into HONE1 and HK1-EBV cells, whereas miR-454-3p mimics were introduced into CNE2 and 5–8F cells. Its overexpression downregulated *PTPRD* mRNA and protein levels in CNE2 and 5–8F cells and elevated *PTPRD* levels in HONE1 and HK1-EBV cells (Figs. 3D, S4A, B).

We performed luciferase reporter assays and found that the luciferase activity of the wt PTPRD 3′-UTR but not of the mutant 3′-UTR was significantly reduced by miR-454-3p mimics but not by the control mimic (NC) (Fig. 3C). To determine the role of miR-454-3p in regulating radiation resistance in NPC, CCK8 assay and colony formation assays were performed. HONE1 and HK1-EBV cells transfected with the miR-454-3p inhibitors were more sensitive to IR than control. Interestingly, when NPC cells transfected with miR-454-3p inhibitors were co-transfected with *PTPRD*-specific siRNA oligos before IR, such sensibility induced by miR-454-3p were partially reduced (Fig. 3E, F, left and Fig. S4C, E). CNE2 and 5–8F cells transfected with miR-454-3p mimics were more resistant to IR than control, and the co-transfection of *PTPRD* plasmids re-sensitized cells to IR (Fig. 3E, F, right and Fig. S4D, F). Collectively, these suggest that miR-454-3p exerts its effects in NPC through the direct suppression of *PTPRD*.

### PTPRD promotes radiation-induced autophagy in NPC cells

**Fig. 4:**
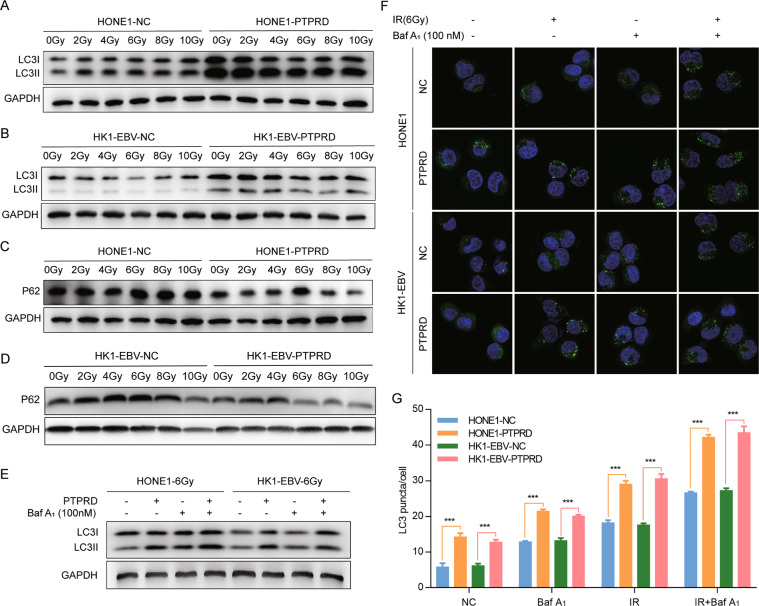
*PTPRD* promotes radiation-induced autophagy in NPC cells. **A–D** Expression of LC3 and P62 was detected in *PTPRD*-overexpressing or control cells after radiation exposure (0–10 Gy) and culture for 24 h subsequently. These data are representative of three independent experiments. **E** Treatment with Baf-A1 caused significant increases in LC3-II accumulation in PTPRD-overexpressing cells after radiation exposure (6 Gy) and culture for 24 h subsequently. These data are representative of three independent experiments. **F**, **G** Immunofluorescence staining images and statistical analysis of LC3 in PTPRD-overexpressing HONE1 and HK1-EBV cells after radiation exposure or Baf-A1 treatment and culture for 24 h subsequently. These data are representative of three independent experiments. Mean ± SD (*n* = 3); Student’s *t* tests; ***P < 0.001.

Gene-set enrichment analysis (GSEA) revealed that *PTPRD* was most strongly associated with autophagy signaling pathways (Fig. S5A). We then determined whether autophagy plays a role in PTPRD-mediated radiation sensitivity. The ratios of LC3-II/LC3-I and LC3-II/actin were increased in *PTPRD*-overexpressing HONE1 and HK1-EBV cells after irradiation (Fig. 4A, B) but were decreased upon *PTPRD*-knockdown in CNE2 and 5–8F cells (Fig. S5B). Consistently, the protein levels of P62 (a substrate of the autophagy pathway) were decreased in *PTPRD*-overexpressing cells but increased in *PTPRD*-knockdown cells, suggesting that radiation-induced autophagy in *PTPRD*-overexpressing NPC cells (Figs. 4C, D, S5B). Statistical analysis of the LC3II/LC3I ratio and P62 protein levels is shown in Fig. S5C–F.

Next, we determined whether the high levels of autophagic markers were due to increased autophagy. As shown in Fig. 4E, treatment with Baf-A1 caused significant increases in LC3-II in *PTPRD*-overexpressing cells, indicating that *PTPRD* can enhance radiation-induced autophagy. Immunofluorescent analysis showed that LC3 signals displayed a punctate membrane pattern in *PTPRD*-overexpressing NPC cells after irradiation (6 Gy), whereas *PTPRD*-knockdown irradiated NPC cells displayed a diffuse cytoplasmic pattern. In addition, treatment with Baf-A1 caused a significantly higher level of membrane LC3 signals than control, indicating autophagosome formation (Fig. 4F, G). Collectively, our results suggest that radiation sensitivity mediated by *PTPRD* is accompanied by an increase in autophagy in NPC cells.

### PTPRD directly targets and dephosphorylates STAT3

**Fig. 5:**
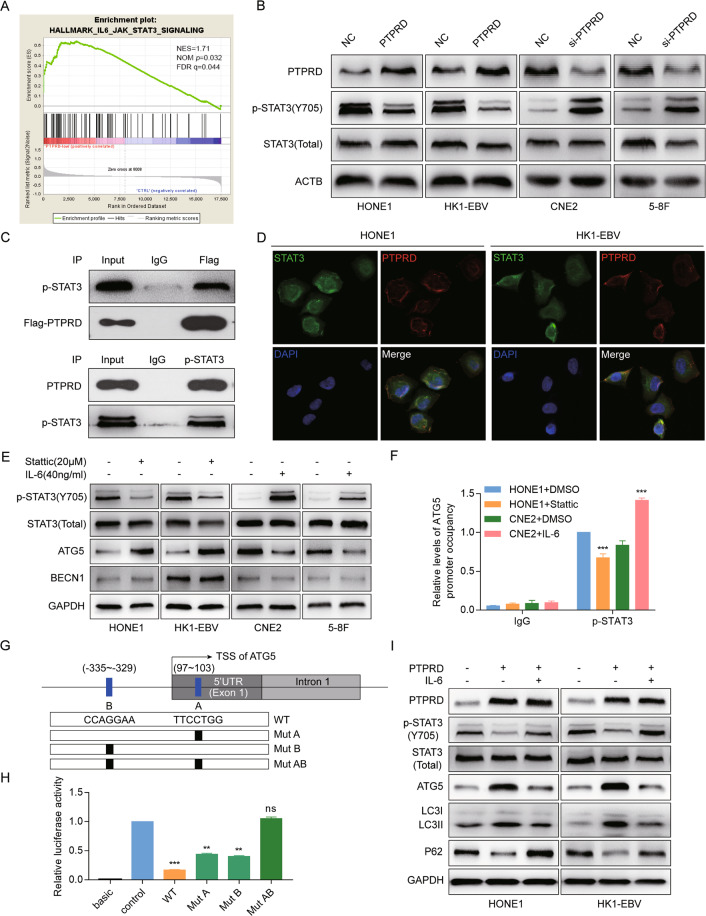
*PTPRD* directly dephosphorylates STAT3 and promotes STAT3-dependent ATG5 transcription. **A** GSEA of the “IL-6/JAK/STAT3 signaling” gene modules in NPC patients with high or low *PTPRD* expression. FDR q false discovery rate q value, NES normalized enrichment score. **B** Western blotting analysis of p-STAT3 and STAT3 protein levels in *PTPRD*-overexpressing or -knockdown cells. These data are representative of three independent experiments. **C** 293T cells treated with IL-6 and co-transfected with flag-tagged *PTPRD* were used for immunoprecipitation. PTPRD and p-STAT3 protein levels were detected via western blotting. These data are representative of three independent experiments. **D** Immunofluorescence staining of ectopically expressed PTPRD and STAT3 in HONE1 and HK1-EBV cells. **E** Expression of *p-STAT3, STAT3, BECN1*, and *ATG5* were detected in NPC cells treated with stattic or IL-6. GAPDH was used as an internal control. **F** ChIP analysis for detection of p-STAT3 binding to the ATG5 promoter in HONE1 and CNE2 cells after treatment with Stattic (20 Μm) and IL-6 (40 ng/mL) respectively. These data are representative of three independent experiments. Mean ± SD (*n* = 3); Student’s *t* tests; ***P < 0.001. **G** Schematic illustration of the 2-kb region upstream of the transcription start site of ATG5. Two potential binding sites of STAT3 at −335 to −329 and 97–103 were identified and labeled as A and B, respectively. **H** 293T cells transfected with the wild-type or mutant pGL4-ATG5 promoter were treated with IL-6 and subjected to dual-luciferase reporter assays. These data are representative of three independent experiments. Mean ± SD (*n* = 3); Student’s *t* tests; **P < 0.01; ***P < 0.001. **I** HONE1 and HK1-EBV cells stably expressing *PTPRD* or the control vector were treated with IL-6 and exposed to radiation. The protein levels of PTPRD, p-STAT3, STAT3, ATG5, LC3I/II, and P62 were detected using western blotting. GAPDH was used as an internal control. These data are representative of three independent experiments.

To explore the potential pathway involved in PTPRD regulation network, GSEA of NPC patients’ expression profiles (GSE12452) was performed. We found that low *PTPRD* expression levels in NPC positively correlate with the Jak/STAT3 signaling pathway (Fig. 5A). Consistent with this, *PTPRD* overexpression inhibited STAT3 phosphorylation, whereas *PTPRD* knockdown activated the expression of phosphorylated STAT3 (p-STAT3; Y705) in NPC cells (Fig. 5B). The endogenous interaction between *PTPRD* and p-STAT3 was demonstrated by coimmunoprecipitation in 293T cells (Fig. 5C). Immunofluorescence staining showed the colocalization of ectopically expressed *PTPRD* and STAT3 in HONE1 and HK1-EBV cells (Fig. 5D). Taken together, this indicates that *PTPRD* might function through STAT3 binding and dephosphorylation.

### PTPRD promotes STAT3-dependent ATG5 transcription

Bioinformatics analysis using the Gene Transcription Regulation database (http://gtrd.biouml.org) showed that autophagy-related genes, including *ATG5* and *BECN1*, might be the potential target genes of STAT3. Following the inhibition of STAT3 phosphorylation by a selective inhibitor, Stattic, we observed that *ATG5* was increased but *BECN1* was not altered using western blotting assays. In contrast, when we enhanced the phosphorylation of STAT3 using IL-6, the protein levels of *ATG5* were decreased but not those of *BECN1* (Fig. 5E). Together, these results showed that *ATG5* exhibited significant changes in all investigated cell lines.

To further investigate the role of p-STAT3 in ATG5 expression, we performed a chromatin immunoprecipitation (ChIP)-PCR assay for detection of p-STAT3 binding to ATG5 promoter. Following the inhibition of STAT3 phosphorylation in HONE1 cells, the enrichment of the ATG5 promoter region was substantially decreased. In contrast, when we enhanced the phosphorylation of STAT3 in CNE2 cells, the enrichment of the ATG5 promoter region was increased (Fig. 5F). Regarding the transcriptional regulatory mechanisms of *ATG5* expression, we identified two potential binding sites of STAT3 at −335 to −329 and 97–103 inside the *ATG5* promoter region. These two transcription factor-binding sites (TFBSs) were named A and B, respectively (Fig. 5G). We then identified the elements responsible for STAT3-mediated suppression of *ATG5*. The luciferase activities of the wild-type ATG5 promoter reporter gene decreased significantly following the enhancement of p-STAT3 (Y705) expression. The activities of the mutated A and B reporter genes were partly decreased, whereas these were not affected after the activation of STAT3 signaling, suggesting both sites A and B are necessary for promoter activity (Fig. 5H).

**Fig. 6:**
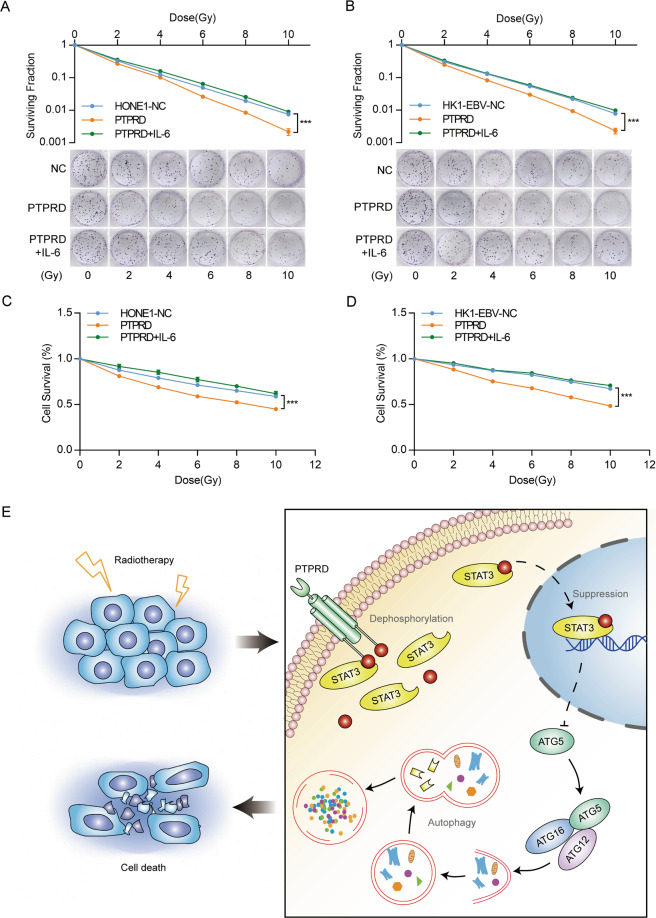
STAT3 activation could rescue the effects of *PTPRD*. **A**–**D** colony formation and CCK8 assays were performed to evaluate cell survival after exposure to the indicated radiation dose (0, 2, 4, 6, 8, and 10 Gy). These data are representative of three independent experiments. Mean ± SD (*n* = 3 and *n* = 5 respectively); two-way ANOVA and Tukey multiple comparison tests; ***P < 0.001. **E** Schematic of the PTPRD-STAT3-ATG5 signaling pathway. PTPRD sensitizes NPC cells to radiotherapy by directly dephosphorylating p-STAT3, thus enhancing ATG5 transcription and resulting in radiation-induced autophagy.

We then determined whether STAT3 activation could rescue the effects of *PTPRD*. The reactivation of STAT3 abrogated increased *ATG5* and LC3 conversion caused by *PTPRD*, whereas the expression of P62 inhibited by *PTPRD* was substantially increased (Fig. 5I). Further, p-STAT3 reactivation significantly abolished the inhibitory effects of *PTPRD* on NPC clonogenic potential (Fig. 6A, B) and cell survival (Fig. 6C, D) after irradiation. Concomitant treatment with Stattic before irradiation rescue the radioresistance effects caused by PTPRD-knockdown expression inversely (Fig. S6). These results suggest that the *PTPRD*-mediated regulation of the STAT3–ATG5–LC3 signaling pathway is one of the major mechanisms underlying the induction of radiation sensitivity. The proposed mechanism is summarized in Fig. 6E.

### PTPRD promotes NPC cells radiation sensitivity in vivo

**Fig. 7:**
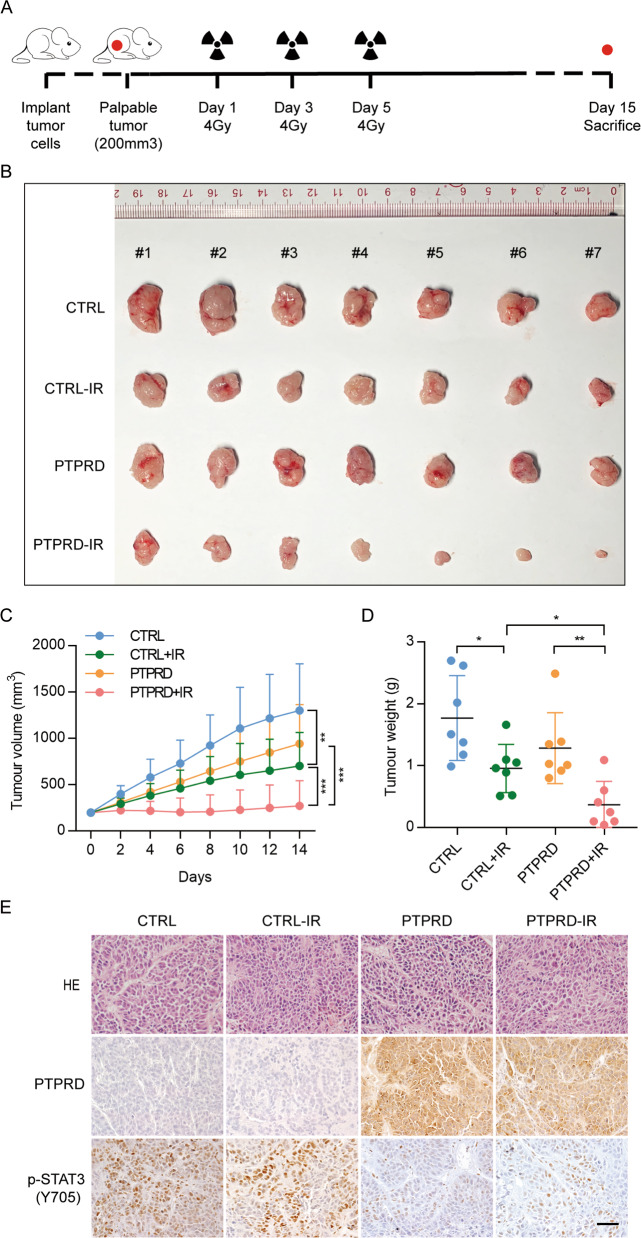
*PTPRD* promotes radiation sensitivity in NPC cells in vivo. **A** Schematic of the in vivo experiment. HONE1 cells stably overexpressing the vector or *PTPRD* were injected subcutaneously into the right flank of male nude mice. Once palpable tumors reached a volume of ~200 mm^3^, mice were subjected to radiation on days 1, 3, and 5 at a dose of 4 Gy. At day 15, the mice were euthanized, and the tumors were isolated, weighed, and compared using ANOVA. **B** Representative images of xenografts from the indicated treatment groups. **C** Tumor growth curves. Mean ± SD (*n* = 7); two-tailed *t* test; **P < 0.01; ***P < 0.001. **D** Tumor weight was measured at the end of the experiment. Mean ± SD (*n* = 7); *P < 0.05; **P < 0.01. **E** Representative HE and IHC staining images.

We determined whether PTPRD sensitizes NPC to radiation in vivo (Fig. 7A). As shown in Fig. 7B, RT moderately suppressed HONE1-CTRL tumor growth compared with non-RT–treated controls (*P* < 0.01) while prominently inhibiting the growth of HONE1-*PTPRD* tumors (*P* < 0.001). Consistent with our in vitro results, HONE1-CTRL xenografts were apparently more resistant to radiation than HONE1-PTPRD xenografts (*P* < 0.001). The average tumor volume and weight in each treatment group at the endpoint exhibited a similar tendency (Fig. 7C, D). The p-STAT3 (Y705) levels were further decreased in *PTPRD*-overexpressing tumors, confirming the results of in vitro experiments (Fig. 7E). Statistical analysis of the PTPRD and p-STAT3 protein levels is shown in Fig. S7. These results indicate that *PTPRD* resensitizes HONE1 xenografts to radiation.

### PTPRD downregulation is associated with poor prognosis in NPC patients

**Fig. 8:**
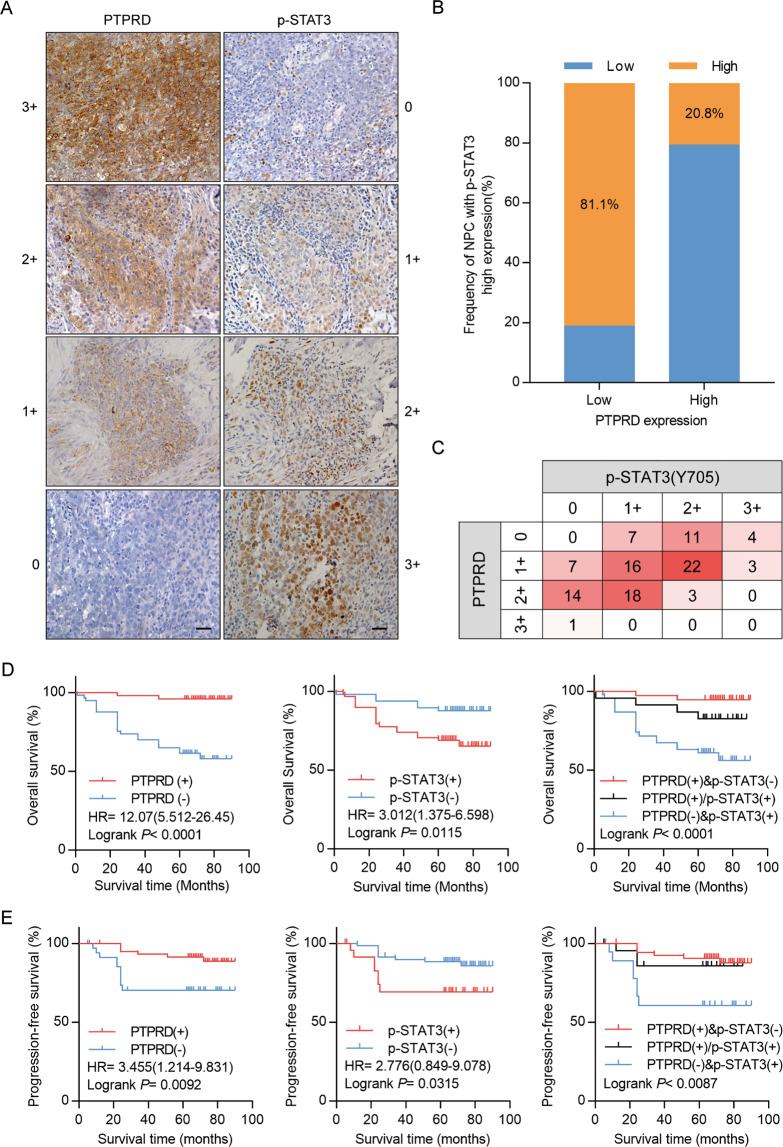
Downregulation of *PTPRD* is associated with poor prognosis in NPC patients, whereas p-STAT3 is a companion biomarker. **A** NPC paraffinized sections (200×, 400×) were used for IHC of the protein levels of PTPRD and p-STAT3. Scale bar: 100 μm. **B**, **C** Percentage of patients showing low or high p-STAT3 protein levels in relation to *PTPRD* expression. **D**, **E** Correlation of the expression levels of *PTPRD* and p-STAT3 with the prognosis of NPC patients. Kaplan–Meier analyses for OS and PFS of NPC patients based on *PTPRD*, p-STAT3 expression, or coincident *PTPRD* and p-STAT3 expression. Log-rank test was used to calculate the *P* values.

We performed immunohistochemical staining of *PTPRD* and p-STAT3 in 107 NPC samples, which was scored between 0 and 3+ according to staining density and extent (Fig. 8A). The clinical characteristics of the NPC patients are summarized in Table S1. Elevated *PTPRD* expression was significantly correlated with TNM stage, mortality, distant metastasis, and recurrence. No significant correlations between *PTPRD* expression and patient age, gender, and World Health Organization’s pathologic type were found. Further, *PTPRD* expression was inversely correlated with p-STAT3 expression (*r* = −0.307, *p* = < 0.001; Fig. 8B, C, Table S2). Kaplan–Meier analysis showed that low expression of *PTPRD* correlates with poor OS and PFS, whereas high p-STAT3 (Y705) level significantly correlates with shorter OS and PFS. Furthermore, NPC patients with higher p-STAT3 (Y705) expression and lower *PTPRD* expression exhibited poorer clinical prognosis (Fig. 8D, E). Multivariate Cox regression analyses revealed that *PTPRD* expression is an independent prognostic factor for OS (Table S3). These results suggest that *PTPRD* can be an independent prognostic predictor for NPC patients.

## Discussion

RT remains the standard treatment regimen for NPC, with almost all of the NPC patients receiving RT during their disease course [22]. Radioresistance is a pressing challenge that leads to treatment failure, and the lack of radiotherapy-specific predictive biomarkers is a major clinical problem. In this study, we identified *PTPRD* as a radiotherapy sensitizer for NPC both in vitro and in vivo. Mechanistically, we demonstrated that *PTPRD* overexpression sensitizes NPC cells to radiotherapy by regulating autophagy-related cell death through the direct targeting of STAT3, whereas the reactivation of STAT3 abrogated the effects of *PTPRD* overexpression. Furthermore, patients with low *PTPRD* expression and high STAT3 phosphorylation exhibited poor OS and PFS.

Epigenetic alterations, including abnormally expressed miRNA and aberrant DNA methylation, can regulate gene expression without altering the nucleotide sequence [23, 24]. They play an important role in the initiation, progression, and treatment resistance of NPC. Based on the two microarray data sets, we identified *PTPRD* as a candidate gene that was downregulated by miRNA and hypermethylated in its promoter, leading to epigenetic silencing in NPC. On the one hand, miR-454-3p directly interacts with 3′ UTR of PTPRD mRNA to negatively regulate *PTPRD* expression, and further to regulate radiation sensitivity in NPC cells. The reversal of miR-454-3p expression also rescues the biological effect of *PTPRD* in NPC cells. On the other hand, we confirmed the hypermethylated status of *PTPRD* in NPC tissues and NPC cell lines using bisulfite pyrosequencing analysis. Accumulating evidence demonstrates the widely aberrant DNA methylation patterns in NPC. The hypermethylation of *SHISA3* and *NFAT1* promotes metastasis in NPC [25, 26]. whereas that of *PCDH17* promotes cell proliferation, migration, and angiogenesis [27]. In addition, previous studies have demonstrated that the hypermethylation of *HOPX, RAB37*, and *ARNTL* enhances the chemosensitivity of NPC cells [28–30]. This brings us to a question. Is aberrant DNA methylation or miR-454-3p plays a dominant role in PTPRD expression and function? As is showed in Fig. S8, we found that both inhibition of miR-454-3p and demethylation of PTPRD could increase PTPRD protein levels, concomitant treatment with miR-454-3p inhibitor, and demethylation drug Dac resulted in higher expression of PTPRD. In addition, transfection with the miR-454-3p mimics could partly abrogate the upregulation of PTPRD by treatment with DAC. Collectively, these suggested that aberrant DNA methylation and miR-454-3p can regulate PTPRD expression synergistically. Which one plays a dominant role may depend on the expression levels of miRNA and the methylation levels of DNA in particular individuals.

Understanding the molecular mechanisms underlying radioresistance in NPC cells is essential for improving the efficacies of RT. RT causes cell death by triggering responses to cellular stresses, including the generation of reactive oxygen species, DNA damage response, membrane lipid peroxidation, mitochondrial damage, endoplasmic reticulum stress, and autophagy [31]. However, the activation of the damage-repair signaling pathways and the inhibition of death-related signaling pathways in cancer cells are responsible for radioresistance [32–34]. Growing evidence suggests that autophagy can enhance the sensitivity of radiotherapy by inducing autophagic cell death. It has been reported that DDR1 inhibition sensitizes glioblastoma cells to radiotherapy by inducing autophagy [35]. The inhibition of mTOR by RAD001 or rapamycin enhances radiosensitization via the induction of autophagy in non-small cell lung cancer in vivo [36]. Consistent with the above reports, our results suggest that *PTPRD* promotes radiation-induced autophagy in NPC cells, resulting in radiosensitization and eventual cell death of NPC cells. Therefore, *PTPRD* may be a potential target for eliminating resistance to radiotherapy in NPC.

The activation of STAT3, a major transcription factor, has been confirmed to play an important role in promoting cell growth, migration, invasion, and chemoresistance in many types of malignant tumors, including pancreatic cancer, breast cancer, head and neck cancer, ovarian cancer, and lung cancer [37, 38]. Therefore, elucidating the molecular mechanisms that regulate STAT3 may contribute to the development of effective anticancer therapies. Evidence exists that p-STAT3 is mainly negatively regulated by PTPs, enzymes that dephosphorylate the tyrosine residue in phosphotyrosine proteins. However, the relationship between STAT3 and *PTPRD* in NPCremains unclear. Our findings suggest that p-STAT3 can be a functional target of *PTPRD* in NPC as supported by the following observations: (a) GSEA showed the significant enrichment of the Jak/STAT3 signaling pathway in NPC patients with low *PTPRD* expression, (b) Western blotting and IHC assays indicated a negative correlation between *PTPRD* and p-STAT3 in vivo and in vitro, and (c) coimmunoprecipitation and immunofluorescence assays demonstrated that *PTPRD* directly interacts with p-STAT3. Interestingly, previous studies have reported that 7 PTPs, namely PTPRD, PTPRT, PTPRK, SHP1, SHP2, MEG2, and TC-PTP, have been implicated in the regulation of STAT3 [5]. It is possible that other tyrosine phosphatases can also regulate p-STAT3 levels and compensate for dephosphorylation when *PTPRD* is downregulated. The gene-expression levels and the function of other tyrosine phosphatases in NPC warrant further studies.

Increasing studies have demonstrated that STAT3 functions as important regulator of a number of autophagy-related genes, and that its activity is associated with cell autophagy [39]. STAT3 can inhibit BECN1 by directly repressing its transcription or upregulating the BECN1-negative regulators BCL2 and MCL1 [40, 41]. In addition, STAT3 can directly bind to the promoter of ATF6, activate its transcriptional activity, and subsequently promote autophagy [42]. STAT3 has also been reported to promote BNIP3 transcription and inhibit the mTOR signaling pathway, thus inhibiting autophagy [43]. We are the first to identify ATG5 as a direct target of p-STAT3 and that it mediates the autophagic-promoting effect of *PTPRD* after irradiation.

In conclusion, this is the first study to demonstrate that *PTPRD* confers radiosensitization in NPC cells by promoting radiation-induced autophagy. We further revealed that the induction of autophagy by *PTPRD* is mediated by its impedance on the p-STAT3-mediated ATG5 transcriptional inactivation. Our findings offer several novel therapeutic targets that can be used in combination with radiochemotherapy in NPC patients.

## Materials and methods

### Sample collection

Twelve primary fresh NPC and 12 noncancerous fresh nasopharyngeal samples were obtained from the Nanfang Hospital (Guangzhou, China) at the time of diagnosis before any therapy. All fresh samples were immediately preserved in liquid nitrogen until RNA or DNA extraction. A total of 117 NPC and 50 noncancerous nasopharyngeal specimens, all paraffin-embedded with detailed long-term follow-up clinical data, were obtained between January 1, 2011 and June 30, 2013. All specimens were pathologically confirmed by two pathologists and collected from the Nanfang Hospital (Guangzhou, China) before any antitumor treatment. TNM staging was reclassified according to the American Joint Committee on Cancer 8th edition. Patients have given their informed consent, and all procedures in this study were approved by the Ethics Committee of the Nanfang Hospital, Guangzhou, China.

### Cell culture and reagents

Five EBV-negative NPC cell lines, namely CNE1, CNE2, HONE1, 5–8F, 6–10B, were generously provided by Prof. Musheng Zeng (Sun Yat-sen University Cancer Center, China). The HK1-EBV and two immortalized normal human nasopharyngeal epithelial cell lines NP460hTert-EBV and NP460hTert were provided by Prof. George S.W. Tsao, University of Hong Kong, China. The cell lines tested negative for mycoplasma contamination (Qiagen, Germany). The NPC cell lines were cultured in RPMI-1640 (Invitrogen, USA) supplemented with 10% newborn cow serum (Gibco, USA); NP460hTert-EBV and NP460hTert, defined keratinocyte serum‐free medium (Invitrogen); and 293T cells, DMEM (Invitrogen) supplemented with 10% newborn cow serum (Gibco). The cell lines were incubated in a humidified chamber with 5% CO_2_ at 37 °C. All gifted cell lines were authenticated by STR profile. Barflomycin A1 (Baf-A1; Selleckchem, USA), Stattic (Selleckchem), and IL-6 (Novoprotein, USA) were dissolved according to the manufacturers’ instructions. The antibodies used for subsequent experiments are listed in Table S4.

### Immunohistochemistry (IHC)

IHC was performed on the paraffin-embedded sections of clinical NPC and xenograft mice tissues. Briefly, the specimens were deparaffinized in xylene and rehydrated using an ethanol gradient. The indirect streptavidin-peroxidase method was used, and the expression levels were evaluated using the intensity and extent of staining as previously reported [44].

### RNA Extraction and qRT-PCR

Total RNA was extracted using TRIzol reagent (TaKaRa, Japan), whereas cDNA was synthesized using a PrimeScript RT reagent kit (TaKaRa). qRT-PCR was performed in triplicate using SYBR Premix ExTaq (TaKaRa). GAPDH and RPU6B were used for normalizing the expression of mRNA and miRNA, respectively. The relative gene expression was calculated using the 2^−ΔΔCt^ method. Independent experiments were done in triplicate, and the primer sequences are listed in Table S5.

### DNA extraction and bisulfite pyrosequencing analysis

Genomic DNA was extracted from fresh tissues using a QIAamp DNA Mini kit (Qiagen) according to the manufacturer’s instructions, whereas bisulfite modification of DNA (1–2 μg) was performed using an EpiTect Bisulfite Kit (Qiagen). Genomic DNA from cells was isolated using a EZ1 DNA Tissue Kit (Qiagen). The region identified as differentially methylated according to the array data was selected for interrogation, and the bisulfite pyrosequencing primers were designed using PyroMark Assay Design Software 2.0 (Qiagen). Pyrosequencing reaction and methylation level quantification were performed using the PyroMark Q96 ID System and software (Qiagen). The primer sequences for PCR and sequencing are shown in Table S4.

### Lentivirus infection and cell transfection

A CRISPR/Cas9-based synergistic activation mediator (SAM) system was used to establish the PTPRD stable-expression cell line. The lentiviral particles carrying the CRISPR/Cas9-SAM PTPRD (pLV-hU6-sgRNA-hef1a-dcas9-NLS-VP64-T2A-Puro) and control vectors were constructed by Kidan-Bio (Guangzhou, China). The harvested virus was incubated with NPC cells for 2 days. After, the cells were subcultured and selected with puromycin (2 μg/mL). Infection efficiency was validated using qRT-PCR and western blotting.

The siRNA oligonucleotides targeting PTPRD, miRNA mimics, or inhibitors (miRNA antisense oligonucleotides) were synthesized by GenePharma (Jiangsu, China). The flag-PTPRD and control plasmids were obtained from GeneChem (Shanghai, China). Plasmid DNAs were purified using a TIANprep Mini Plasmid Kit (TIANGEN, China). Transfection experiments were carried out using Lipofectamine 3000 reagent (Invitrogen) according to the manufacturer’s instructions. The cells were collected for further analysis after transfection for 48 h. The sequences abovementioned are listed in Table S6.

### Cell proliferation and colony formation assays

Cell viability was determined using the Cell Counting Kit-8 (CCK-8) assay after exposure to different doses of X-ray IR. NPC cells (1 × 10^3^ cells/well) were seeded in 96‐well plates, exposed to IR with a 6-MV X-ray beam at 2, 4, 6, 8, or 10 Gy, and cultured for 4 days with 24 h collection. The culture medium was replaced with 100 μL RPMI-1640 medium containing 10 μL CCK‐8 solution. Absorbance was measured on a microplate reader (Bio-Rad) at 450 nm. Five replicates of each treatment were performed, and each experiment was conducted in triplicate. Cell survival was calculated using the following formula: survival rate (%) = OD/OD 0 h × 100%.

Colony formation assays were performed to determine the cellular response to radiation as described previously [45]. Briefly, the transfected cells were seeded at a density of 200, 400, 800, 1600, 3200, or 6400 cells per well in 6-well plates and exposed to 0, 2, 4, 6, 8, or 10 Gy (2 Gy per fraction), respectively, with a 6-MV X-ray beam from an Elekta linear accelerator (Precise 1120; Elekta Instrument AB, Stockholm, Sweden) at a dose rate of 220 cGy/min. After 7–10 days of incubation, the colonies were fixed with 4% paraformaldehyde and stained with 0.5% crystal violet. Colonies containing more than 50 cells were counted, and the surviving fraction was calculated. All experiments were performed thrice.

### Luciferase reporter assays

The 3′-UTR sequences of PTPRD containing the wild-type or mutant predicted binding sites of has-miR-454-3p were cloned into the pGL4 vector. 293T cells transfected with miRNA mimics or control were seeded and co-transfected with pGL4 and pRL-TK vector using Lipofectamine 3000 reagent (Invitrogen). After 48 h, the luciferase activity was measured using a Dual Luciferase Assay kit (Promega).

Luciferase reporter assays were also performed to identify the interaction between the transcription factor STAT3 and the ATG5 promoter. The luciferase reporter plasmids (Promega) that contained the wild-type and mutant of the ATG5 promoter were constructed. For the reporter assays, wild-type, mutant, or pGL4-control vector was co-transfected with the pRL-TK Renilla vector into 293T cells after subsequent IL-6 treatment. Luciferase activity was measured at 48 h after transfection.

### Coimmunoprecipitation

The transfected cells were lysed with IP lysis buffer (Beyotime Biotechnology, USA) containing a protease inhibitor cocktail (Roche) and phosphatase inhibitors (Roche). Primary anti-Flag, anti-STAT3, or anti-IgG (negative control) antibodies were incubated with the lysates overnight at 4 °C. Protein A/G Sepharose beads (Santa Cruz, USA) were added to the immune-complexes for recovery. After washing three times with PBS to remove unbound proteins, the cells were suspended in 2 × SDS-sample buffer and boiled for 10 min. Western blotting was then performed.

### In vivo xenograft tumor models

The animal procedures in this study were approved by the Ethical Committee for Animal Research of Southern Medical University (Guangzhou, China) and were performed to minimize animal suffering. The nude mice (3–4 weeks old, male) were purchased from the Central Animal Facility of the Southern Medical University. HONE1 cells (1 × 10^7^ cells in 100 μL PBS) that stably overexpressed the vector or PTPRD were injected subcutaneously into the right flank of the mice. Tumor volume was calculated using the formula: volume = [length × (width^2^)]/2. Mice were randomly assigned to treatment groups (seven mice per group). When palpable tumors reached a volume of ~200 mm^3^, the mice were subjected to radiation with an Elekta 6-MV photon linear accelerator. Before IR, each mouse was anesthetized with pentobarbital (40 mg/kg) and shielded by a lead shield, so that only the xenograft tumor exposed. Radiation treatments were given on days 1, 3, and 5 at a dose of 4 Gy with a dose rate of 1 Gy/min (12 Gy in total). Tumor sizes were monitored for every 2 days for ~2 weeks. Then, the mice were killed, and the tumors were collected for H&E staining and immunostaining analyses.

### Statistical analysis

Data were analyzed using SPSS v19.0 and GraphPad Prism v7.0 (GraphPad Software Inc.) and are presented as the mean ± SEM, unless otherwise specified, of at least three independent experiments. *P* < 0.05 was considered statistically significant. Comparisons between two groups were performed using Student’s *t* test, whereas one-way ANOVA (analysis of variance) was used for multiple group comparisons. The parametric generalized linear model with random effects was used for tumor growth, CCK8 assay, and colony formation assay. The Kaplan–Meier method and univariate analysis were used to estimate the survival curves, and multivariate Cox regression analysis with the backward stepwise method was used to determine the independent prognostic factors. Single, double, and triple asterisks indicate statistical significance (*P < 0.05, **P < 0.01, and ***P < 0.001).

## Supplementary information

Table S1-3

Supplementary Information
