# Supplementary material for: Protein tyrosine phosphatase receptor type D gene promotes radiosensitivity via STAT3 dephosphorylation in nasopharyngeal carcinoma
Source: Oncogene. 2021 Apr 6;40(17):3101–17. doi: 10.1038/s41388-021-01768-8 (PMC8084736; doi:10.1038/s41388-021-01768-8)
Supplement: Supplementary file 2 — Supplementary Information [file 41388_2021_1768_MOESM2_ESM.pdf]

Supplementary Information

Supplementary Table S4. A list of antibodies used for WB, IHC, IFC, IP.

Antigen	Catalog number	Source	Application&Dilutions
PTPRD	BS71853	Bioworld	WB (1:1000); IHC/IFC (1:200)
LC3	14600-1-AP	Proteintech	WB (1:1000)
P62/SQSTM1	18420-1-AP	Proteintech	WB (1:1000)
STAT3 (Y705)	ab76315	abcam	IHC/IFC (1:100); IP (1:200)
STAT3 (Y705)	9145	CST	WB (1:1000)
STAT3	10253-2-AP	Proteintech	WB (1:1000)
ATG5	12994	CST	WB (1:1000)
BECN1	3495	CST	WB (1:1000)
Flag	2064	DIA-AN	IP (1:200)
IgG	B900640	Proteintech	IP (1:200)
GAPDH	381918	ZEN-BIO	WB (1:1000)
β-actin	FD0060	Fdbio Science	WB (1:7500)

Supplementary Table S5. The primers used in this study.

Primers name		Primer sequence
PTPRD	Forward	CTCAGCAGCTCGTGCCTGTAATC
	Reverse	CACTGGTGCGATCATGGCTTACTG
CYLD	Forward	CTGGAGTTGTACGCTTCAGAGGAC
	Reverse	ACCACGACCTTCTTCCAGCAATTC
FRZB	Forward	TTGCTTCTTGTGACTGCTCCTTCC
	Reverse	GCATTCCATTCCTCCAGGCATCC
CUL3	Forward	TGGCTCTTGGCTAGGCTAGGTG
	Reverse	GGCTGTGCTGTGACTGTGACTC
KLF11	Forward	GAGGCAGGAGAATCGCTTGAACC
	Reverse	TCCAGCACTCCACGCAGGTAG
TFAP2B	Forward	ATGCCAATGACAACGACACTGAGG
	Reverse	CGTGTGCCTGTTAGTGGTGGTG
β-actin	Forward	TCAAGATCATTGCTCCTCCTGA
	Reverse	CTCGTCATACTCCTGCTTGCTG
Has-miR-454-3p		cggcgTAGTGCAATATTGCTTATAGGGT
RPU6B		CTCGCTTCGGCAGCACATATA
Bisulfite-PTPRD	Forward	GGTTTTAGGAGAGAAGTGAATTTAGTA
	Reverse	CCCCATATCTTCCCCTCTTA（5’-Biotin）
pyrosequencing		TTGTTAGAGGAGGTTTAGAG

Supplementary Table S6. Sequences used in this study.

gene	NO.		sequence
PTPRD siRNA	1	Sense	5’ CUGGAUCAGUUCUCAGAAUTT 3’
		Antisense	5’ AUUCUGAGAACUGAUCCAGTT 3’
	2	Sense	5’ CACCCACUAAUCAUGAAAUTT 3’
		Antisense	5’ AUUUCAUGAUUAGUGGGUGTT 3’
	3	Sense	5’ GCAGCCACUAAACUUCAAATT 3’
		Antisense	5’ UUUGAAGUUUAGUGGCUGCTT 3’
	4	Sense	5’ CCUUCGGACACUACCAAAUTT 3’
		Antisense	5’ AUUUGGUAGUGUCCGAAGGTT 3’
miR-454-mimics		Sense	5’ UAGUGCAAUAUUGCUUAUAGGGU 3’
		Antisense	5’ CCUAUAAGCAAUAUUGCACUAUU 3’
Negative control		Sense	5’ UUCUCCGAACGUGUCACGUTT 3’
		Antisense	5’ ACGUGACACGUUCGGAGAATT 3’
miR-454-inhibitor		Sense	5’ ACCCUAUAAGCAAUAUUGCACUA 3’

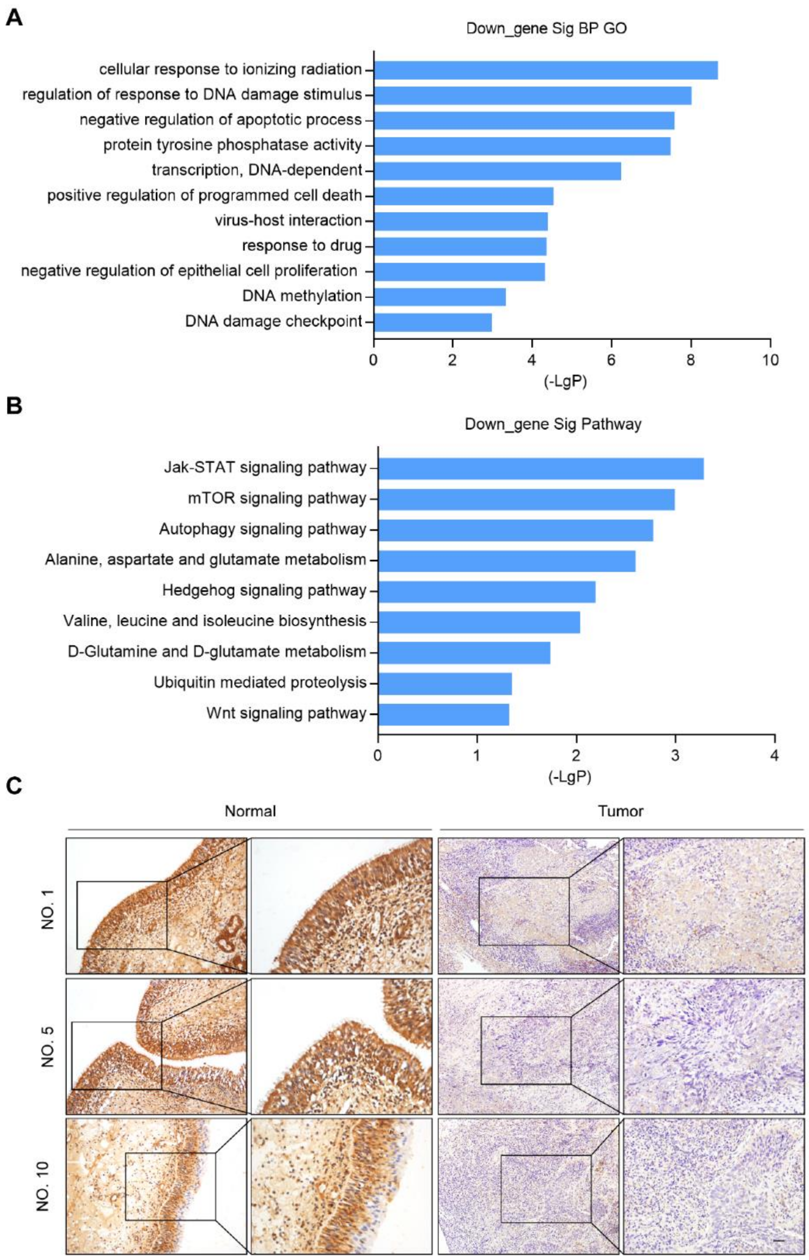

Supplementary Figure S1. (**A**) GO functional analysis revealed that candidate genes were involved in cellular response to ionizing radiation. (**B**) KEGG pathway analysis evealed that candidate genes were involved in Jak/STAT pathway. (**C**) Representative IHC staining images of PTPRD in 117 NPC tissues and 20 NP tissues. Scale bar: 100mm.

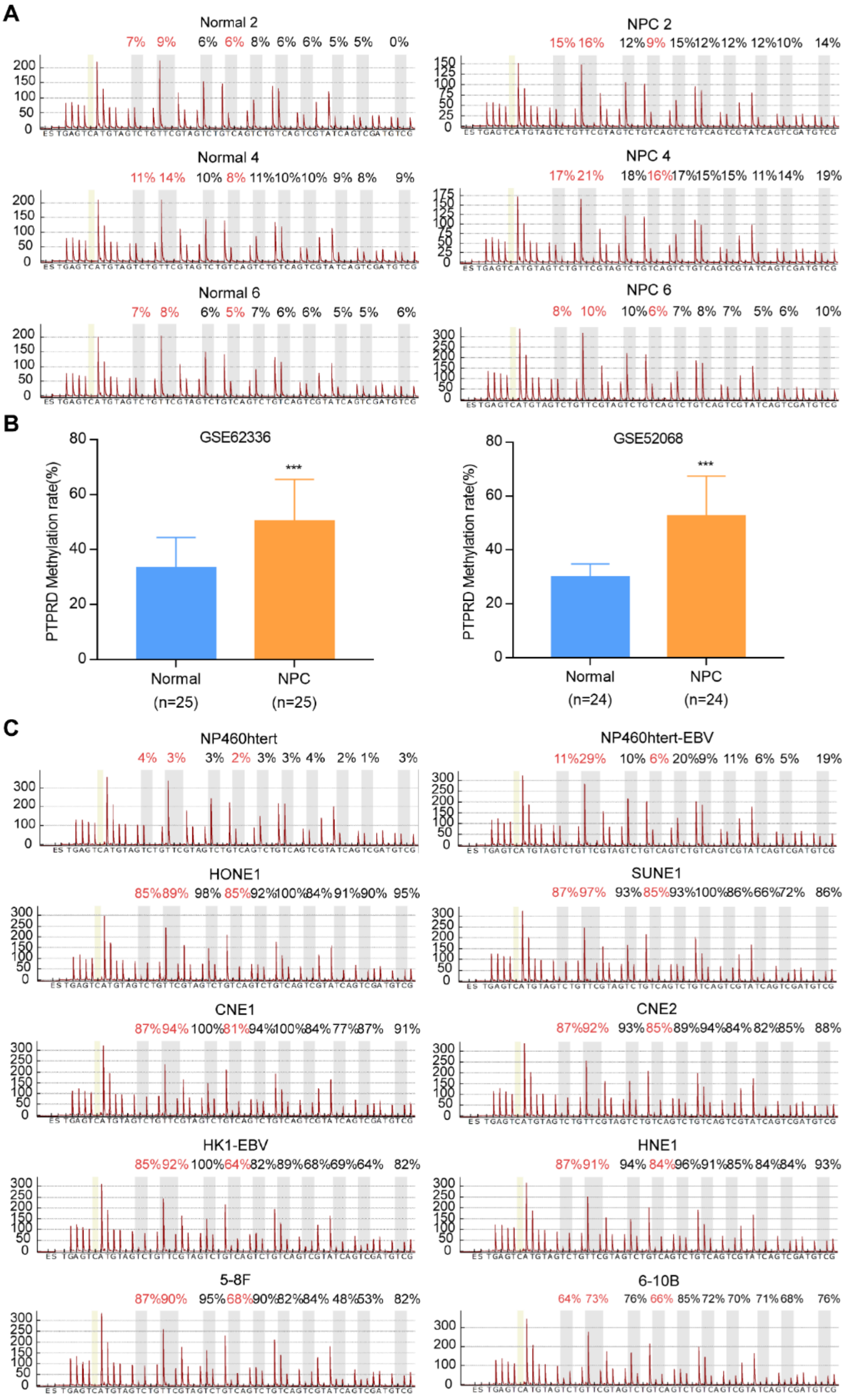

Supplementary Figure S2. (**A**) Bisulfite pyrosequencing analysis of the PTPRD promoter region in NPC and NP tissues. (**B**) Genome-wide methylation microarray data downloaded from GEO (GSE62336 and GSE52068) were reanalyzed to identify the methylation level of CG site (cg14080967) in the promoter region of PTPRD. Mean ± SD; Student's t-tests; ***P<0.001. (**C**) Bisulfite pyrosequencing analysis of the PTPRD promoter region in NPC and NP cell lines.

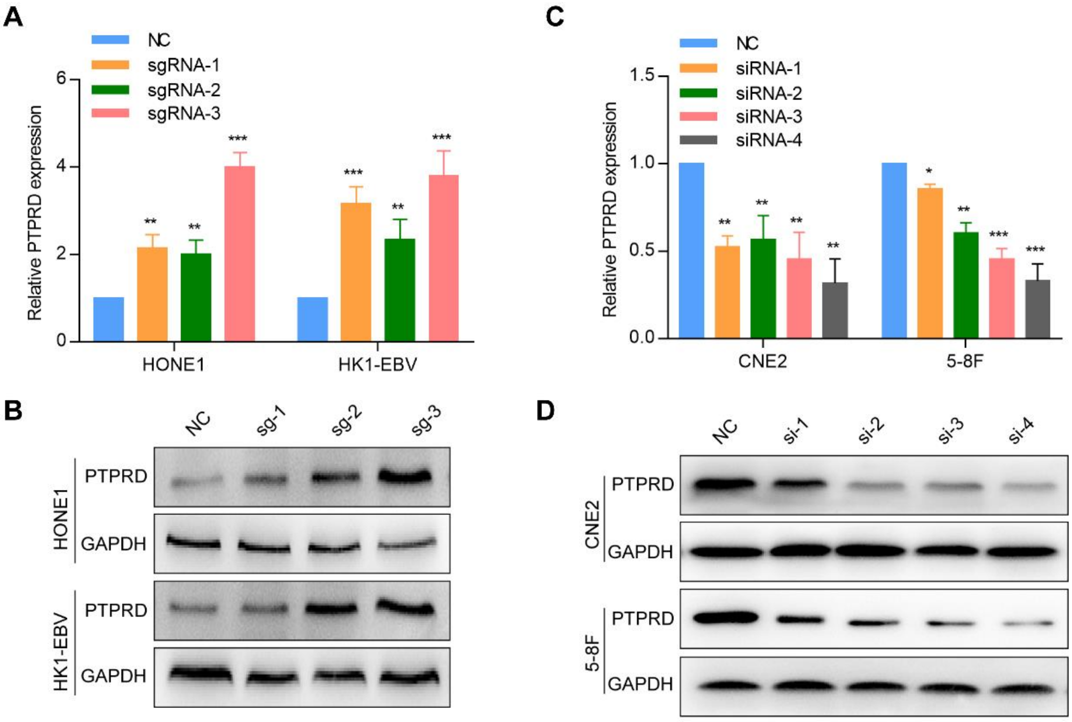

Supplementary Figure S3. Overexpression and knockdown efficiency of PTPRD. (**A, B**) Lentivirus infection efficiency of PTPRD was detected by qRT-PCR assays and western blot assays. Three sgRNAs were designed to target PTPRD for CRISPR/Cas9-based synergistic activation. These data are representative of three independent experiments. Mean ± SD (n=3); Student's t-tests; **P*<0.05, ***P*<0.01, ****P*<0.001. (**C, D**) Transfection efficiency of PTPRD-specific siRNA oligos was detected by qRT-PCR assays and western blot assays. Mean ± SD (n=3); Student's t-tests; **P*<0.05, ***P*<0.01, ****P*<0.001.

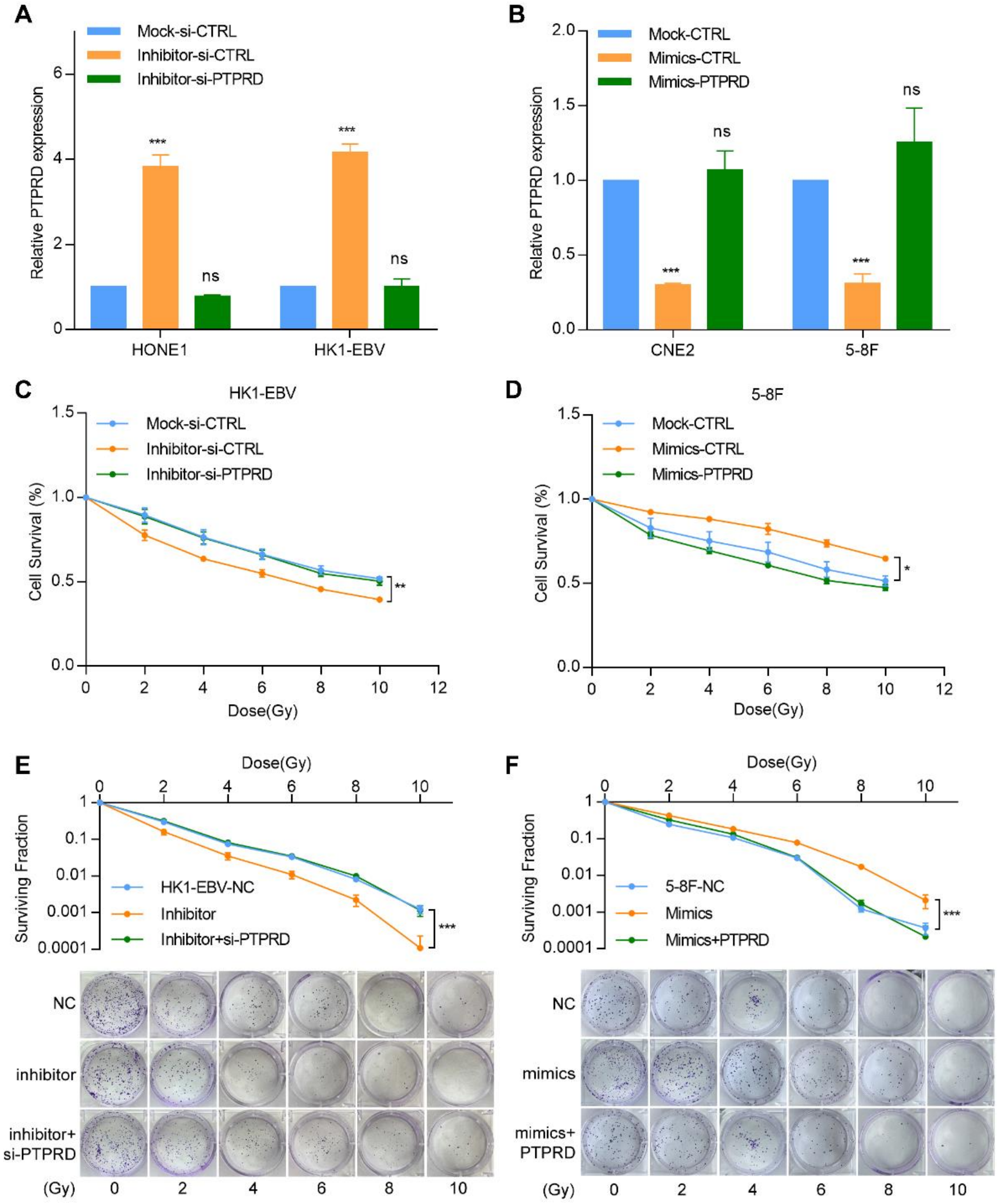

Supplementary Figure S4. (**A, B**) qRT-PCR of PTPRD in NPC cells following transfection with miR-454-3p mimics/inhibitor or co-transfection with PTPRD-specific plasmid /siRNA. These data are representative of three independent experiments. Mean ± SD (n=3); Student's t-tests; ****P*<0.001. (**C, D**) CCK8 assay and (**E, F**) colony formation assay were performed to evaluated cell survival after exposure to the indicated radiation doses (0, 2, 4, 6, 8, 10 Gy). These data are representative of three independent experiments. Mean ± SD (n=5 and n=3 respectively); two-way ANOVA and Tukey multiple comparison tests; **P*<0.05, ***P*<0.01, ****P*<0.001.

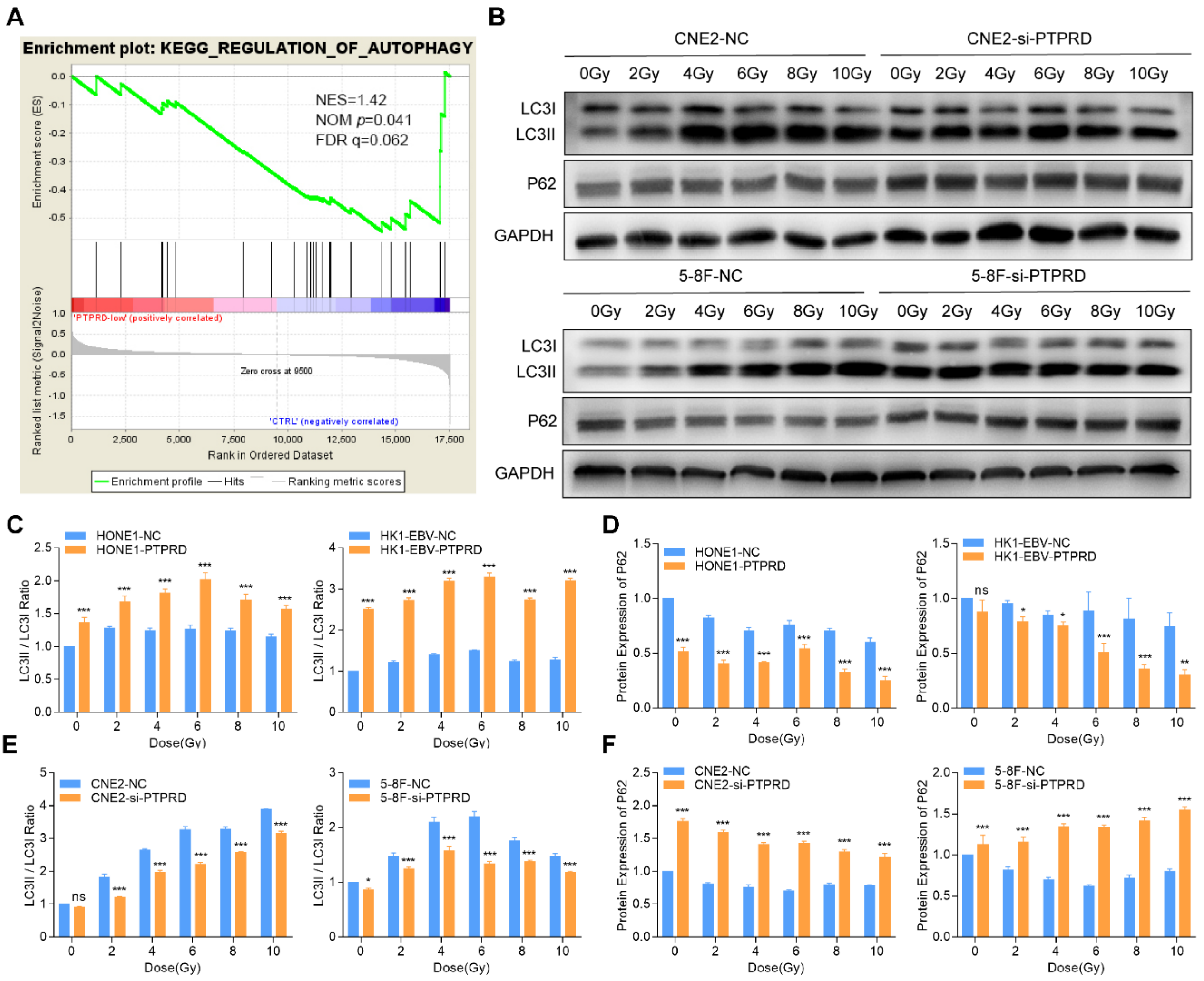

Supplementary Figure S5. (**A**) GSEA analysis of the "Regulation of autophgy" gene modules in NPC patients with high or low PTPRD expression. FDR q false discovery rate q value, NES normalized enrichment score. (**B**) Expression of LC3 and P62 were detected in PTPRD-knockdown or control cells after exposed to radiation (0–10 Gy). These data are representative of three independent experiments. (**C-F**) Statistical analysis of LC3II/LC3I ratio and P62 protein levels in western blotting. These data are representative of three independent experiments. Mean ± SD (n=3); two-way ANOVA and Tukey multiple comparison tests; *P<0.05, **P<0.01, ***P<0.001.

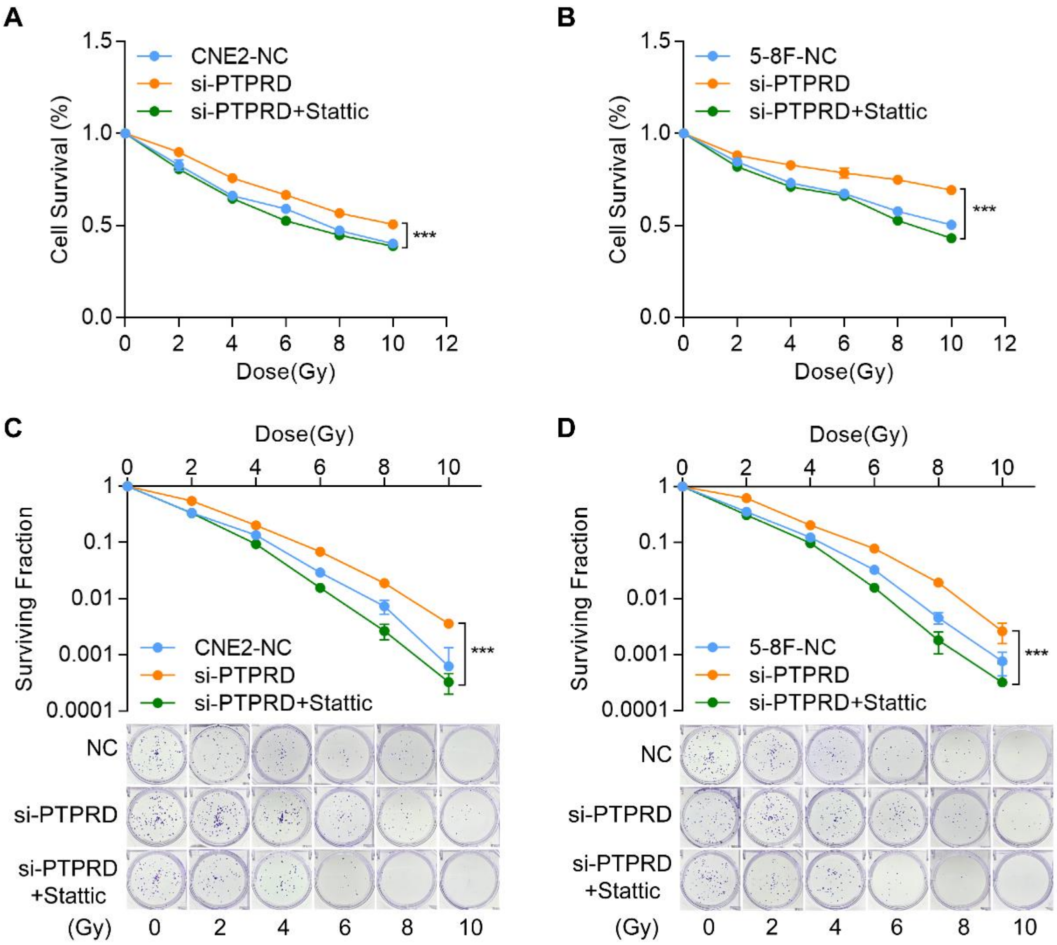

Supplementary Figure S6. (**A, B**) CCK8 and (**C, D**) colony formation assays and were performed to evaluate cell survival after treatment with stattic (0.1μM, 24h) and subsequently exposure to the indicated radiation dose (0, 2, 4, 6, 8, 10 Gy). These data are representative of three independent experiments. Mean ± SD (n=5 and n=3 respectively); two-way ANOVA and Tukey multiple comparison tests; ***P<0.001.

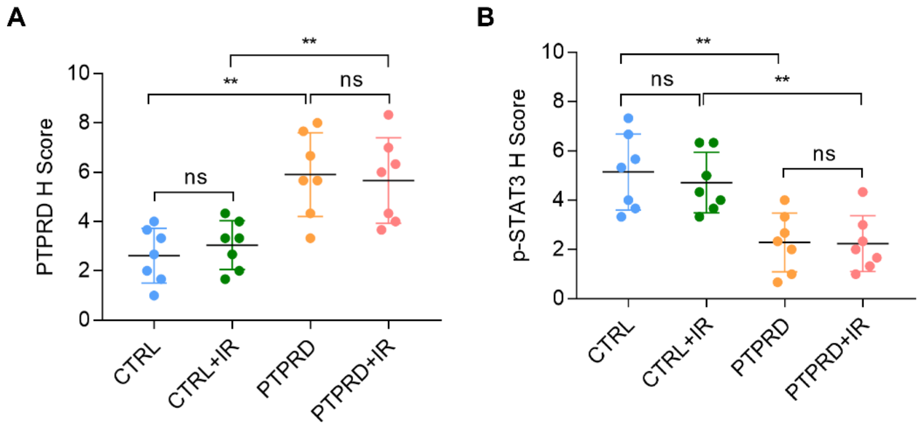

Supplementary Figure S7. (**A**, **B**) IHC statistical analysis of PTPRD and p-STAT3 expression in xenografts tissues. Mean ± SD (n=7); Student's t-tests; ***P<0.001.

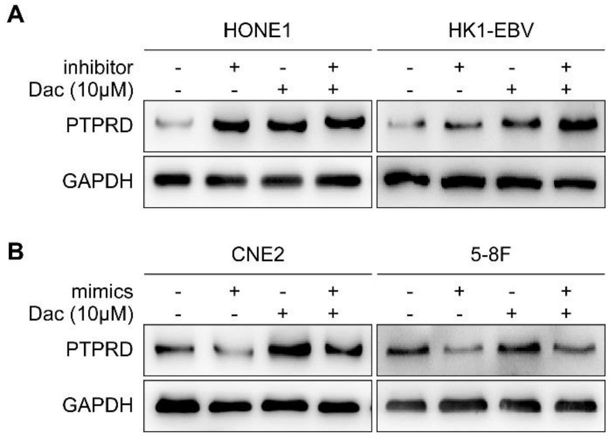

Supplementary Figure S8. (**A**) PTPRD expression was detected following transfection with miR-454-3p inhibitor, treatment with demethylation drug DAC, or co-treatment with inhibitor and DAC in HONE1 and HK1-EBV cells. These data are representative of three independent experiments. (**B**) PTPRD expression was detected following transfection with miR-454-3p mimics, treatment with demethylation drug DAC, or co-treatment with mimics and DAC in CNE2 and 5-8F cells. GAPDH was used as a loading control. These data are representative of three independent experiments.
